# Nutritional counseling strategies in breastfeeding: a scoping review

**DOI:** 10.3389/fnut.2026.1703970

**Published:** 2026-02-24

**Authors:** Lenycia de Cassya Lopes Neri, Monica Guglielmetti, Dalma Chiapponi, Simona Fiorini, Cinzia Ferraris

**Affiliations:** 1 Laboratory of Food Education and Sport Nutrition, Department of Public Health, Experimental and Forensic Medicine, University of Pavia, Pavia, Italy; 2 Human Nutrition and Eating Disorders Research Center, Department of Public Health, Experimental and Forensic Medicine, University of Pavia, Pavia, Italy; 3 Private Practitioner, Livorno, Italy

**Keywords:** breastfeeding, exclusive breastfeeding, maternal education, nutritional counseling, scoping review

## Abstract

Breastfeeding provides unparalleled health benefits for infants and mothers, yet exclusive breastfeeding (EBF) rates remain suboptimal worldwide. Nutritional counseling (NC) has been identified as a key strategy to support and sustain breastfeeding, but the scope and effectiveness of such interventions have not been systematically mapped. We conducted a scoping review to identify and synthesize evidence on NC strategies for breastfeeding mothers. Following Arksey & O’Malley’s framework and PRISMA-ScR guidelines, we searched PubMed (2015–2025) for studies of prenatal or postnatal counseling interventions aimed at improving breastfeeding outcomes. Twenty-nine studies (RCTs, quasi-experimental, qualitative) from diverse countries were included. Counseling interventions (delivered individually, remotely or face-to-face) were generally associated with higher EBF rates and longer breastfeeding duration. Twelve studies also reported improvements in maternal breastfeeding self-efficacy and reductions in common breastfeeding problems. Mobile-based and peer support interventions showed promising results, especially in low-resource settings. These findings align with current global recommendations on breastfeeding counseling. We discuss implementation challenges (e.g., training needs, definitional clarity) and underline that targeted NC is an effective tool to increase breastfeeding rates. Future work should standardize counseling approaches, expand training for providers, and evaluate long-term impacts and cost-effectiveness.

## 1 Introduction

Breastfeeding is the optimal source of nutrition for infants, conferring protection against infections, chronic diseases, and mortality (1). The World Health Organization (WHO) and the United Nation Children’s Fund (UNICEF) recommend exclusive breastfeeding (EBF) for the first 6 months and continued breastfeeding up to 2 years or beyond (2). However, global EBF rates remain low, with only about 44% of infants aged 0–6 months being exclusively breastfed (3, 4). Suboptimal breastfeeding contributes to malnutrition, increased morbidity, and poorer developmental outcomes in early childhood (5). One study in low-resource settings reports that nearly half of maternal and child undernutrition, and a significant proportion of child mortality, can be attributed to poor feeding practices, including suboptimal breastfeeding (6). For mothers, breastfeeding reduces risks of breast cancer, diabetes and improves birth spacing (7). Several studies have demonstrated the positive impact of counseling interventions on breastfeeding outcomes. Evidence indicated positive effects of counseling on EBF indicators, and the strategy proved to be a powerful tool for maternal empowerment (8). For instance, Bueno-Gutiérrez et al. reported a 30% increase in EBF at 2 months post-intervention (9). Other studies highlighted improvements in maternal breastfeeding self-efficacy and reductions in common breastfeeding-related problems such as nipple pain, breast engorgement, and perceived insufficient milk supply (2, 5). Mobile-based and peer support interventions have also shown promising results, particularly in low-resource settings. For example, Jerin et al. found that community-based phone support increased five-month EBF rates from 42 to 71% (*p* < 0.001) (10). Recent high-quality evidence further confirms the effectiveness of professional breastfeeding support. A 2025 systematic review and meta-analysis by D’Hollander et al. reported that interventions delivered by lactation consultants significantly reduced the risk of discontinuing exclusive and any breastfeeding and modestly prolonged breastfeeding duration compared with usual care. These findings support the value of specialized counseling within multidisciplinary breastfeeding support models (11). Recognizing these benefits, WHO has set targets to increase EBF to 50% by 2025, and has issued guidelines emphasizing counseling interventions to improve breastfeeding practices (1).

Nutritional counseling (NC) is a client-centered, supportive process that aims to help individuals adopt and maintain healthy dietary behaviors through the integration of nutritional knowledge and communication techniques. It is not limited to the passive transmission of information; rather, it emphasizes building a collaborative relationship between the counselor and the individual, fostering motivation and autonomy (12, 13). Recent literature defines it as a broader interactive process of communication, goal setting, and behavior change support concerning food and nutrition, which can be implemented by various trained health workers. According to Neri and colleagues (14), NC is “a support process characterized by a collaborative interaction through which patients actively participate in the interpretation and management of their nutritional needs and goals.” This broader conceptualization allows the inclusion of different providers, including peer counselors and community health workers, who support mothers through communication-based strategies, even if they are not trained dietitians. This review adopts this broader definition, in line with recent systematic reviews on NC in clinical and community settings (14, 15).

Core strategies of NC include empathetic listening, open-ended questioning, goal-setting, problem-solving, positive reinforcement and, when appropriate, motivational interviewing techniques. These approaches help uncover personal beliefs, emotional and social barriers to change, and contextual factors that influence eating behaviors (15). Counseling can be delivered individually or in groups, face-to-face or remotely, and may involve health professionals or trained peer counselors, depending on the setting and target population. Recent literature has also emphasized the potential of NC from early life stages, including childhood and adolescence, where tailored communication strategies can promote healthy habits and support developmental goals (14). This supports its application even earlier in life, such as during the perinatal period and breastfeeding, as part of a preventive and health-promoting framework.

In the context of maternal and child health, NC plays a critical role, supporting mothers in the overcoming challenges, including breastfeeding, increasing self-efficacy, and addressing misinformation or cultural barriers (1, 16). When implemented with structured strategies and trained personnel, it has been shown to improve both dietary outcomes and broader health indicators (17). NC in breastfeeding combines communication skills with feeding knowledge to support mothers. It typically involves empathetic listening, open-ended discussion, and problem-solving techniques to identify mothers’ concerns and provide tailored advice (5, 18). Counselors may be health professionals (dietitian, nurses, midwives, lactation consultants) or trained peer supporters (women who have successfully breastfed) (19). Interventions often engage family members to build social support. Delivery formats vary widely – from one-on-one sessions and group workshops to home visits, telephone follow-ups, or digital platforms (mobile apps, text messaging) (20). Each strategy aims to improve maternal knowledge, confidence, and self-efficacy regarding breastfeeding. However, there is considerable heterogeneity in how “nutritional counseling” is defined and implemented across studies.

This scoping review aims to chart the existing literature on NC interventions for breastfeeding mothers, during pregnancy or postpartum period. By synthesizing current evidence, we seek to highlight gaps and best practices for supporting breastfeeding through targeted counseling.

## 2 Methods

We conducted a scoping review following Arksey and O’Malley’s framework and the Preferred Reporting Items for Systematic Reviews and Meta-Analyses Extension for Scoping Reviews (PRISMA-ScR) checklist. The protocol for this scoping review was registered in Open Science Framework (OSF) under the DOI: 10.17605/OSF. IO/YTGDB.

### 2.1 Search strategy

We searched the PubMed database for studies published over the last 10 years (2014 through 2025). Search terms included variations of “breastfeeding,” OR “lactation,” AND “counseling,” OR “education” OR “support.” The search was conducted up to March 2025 and updated on 30 December 2025. The PCC mnemonic was used to formulate the review question, with the population (P) defined as women who are breastfeeding or planning to breastfeed, the concept (C) as nutritional counseling procedures, and the context (C) as clinical, social, and cultural situations related to breastfeeding. Based on this framework, the review sought to answer the following question: “What evidence is available in the literature on nutritional counseling for women who are breastfeeding or intend to breastfeed?”

### 2.2 Eligibility criteria

Eligible studies were peer-reviewed articles describing interventions that included nutritional counseling (broadly defined as support or education on breastfeeding and infant nutrition) provided to pregnant or postpartum women. Both quantitative (e.g., randomized trials, quasi-experimental, cohort) and qualitative studies were considered. We excluded reviews, protocols, case reports, and interventions lacking a counseling component.

### 2.3 Study selection and data collection

**Figure 1:**
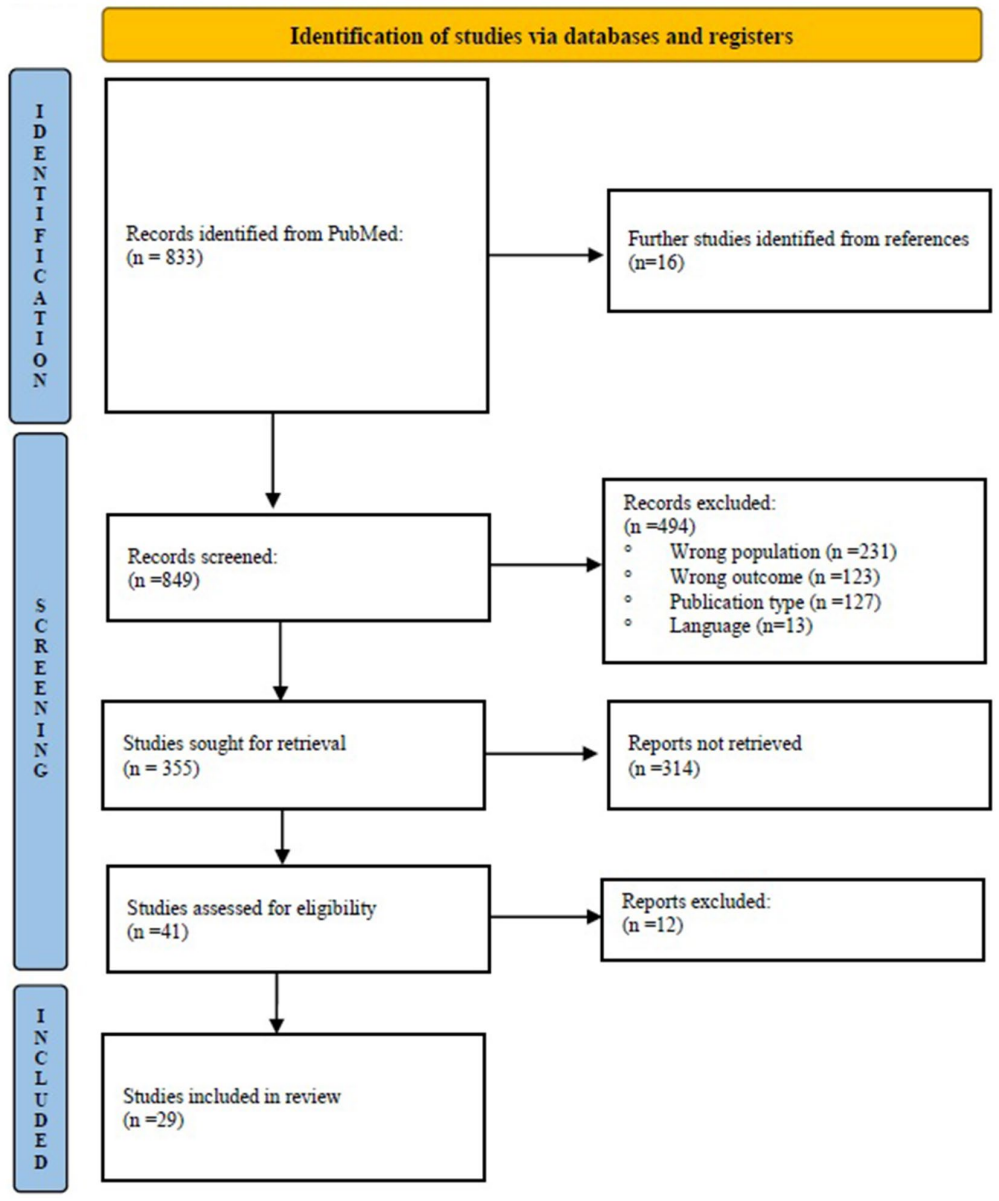
Flow-chart of articles selection.

Two authors (DC and LCLN) independently screened titles and abstracts, then full texts, using Rayyan QCRI (21) to facilitate the process. Discrepancies were resolved by discussion. In total, 849 unique records were identified and screened. After title/abstract review, we retrieved full texts of 41 articles for detailed evaluation. Of these, 29 studies met all inclusion criteria (see PRISMA flow diagram, Figure 1). Common reasons for exclusion were the absence of a breastfeeding counseling element, wrong study design, or interventions focusing solely on general nutrition without breastfeeding support. We extracted data on study characteristics (authors, year, country), design, participant details (sample size, maternal age, infant age), counseling intervention (mode, frequency, provider training), and outcomes (breastfeeding initiation, exclusivity, duration, maternal self-efficacy, infant growth or health). The counseling techniques used (e.g., active listening, motivational interviewing) and any theoretical frameworks cited were extracted. Data were charted in standardized tables to facilitate comparison across studies. No meta-analysis was performed due to heterogeneity in interventions and outcomes; we instead provided a qualitative synthesis of results. As is typical in scoping reviews, we did not conduct a formal risk-of-bias assessment of individual studies, since we aimed to map the evidence rather than exclude studies based on quality.

## 3 Results

We identified 833 records via PubMed and 16 additional studies through manual search of references. After screening and full-text review, 29 studies (published 2015–2025) met inclusion criteria. These were conducted in several countries: 7 in the USA (4, 22–27), 5 in Iran (28–32), 4 in Bangladesh (3, 10, 33, 34), 2 in South Africa (35, 36), 2 in Turkey (6, 37), 2 in the UK (38, 39), 1 in Brazil (40), 1 in Mexico (9), 1 in Spain (41), 1 in India (42), 1 in Denmark (43), 1 in Nigeria (44) and 1 in Australia (45). The interventions often targeted vulnerable groups such as adolescents, low-income mothers, or mothers with preterm infants or HIV. In terms of study design, 16 were randomized controlled trials (RCTs) (3, 6, 9, 26–29, 31, 32, 34–37, 40, 41, 43), 4 qualitative studies (4, 23, 33, 45), 3 quasi-experimental studies (10, 30, 42), 2 pilot feasibility studies (25, 38), 2 intervention studies (22, 44), 1 time-series analysis (39), and 1 secondary analysis of programme data (24). Sample sizes ranged from 28 (4) to more than 5,000 (24).

Interventions differed in setting and delivery format. Five studies (32, 38, 40, 43, 45) were delivered in hospital or maternity wards, 9 (3, 9, 22, 26, 28, 29, 33, 36, 42) in community or primary care clinics, and 15 (4, 6, 10, 23–25, 27, 30, 31, 34, 35, 37, 41, 44) combined hospital counseling with community or remote support.

All included studies were based on one-to-one interventions. Ten (6, 9, 22, 33, 37, 40–44) were conducted by health professionals (nurses, midwives, medical interns), while twelve (3, 4, 23–27, 29, 30, 34, 36, 45) primarily relied on peer counselors (women with prior breastfeeding experience trained to support new mothers) or lactation/breastfeeding specialists. Three studies (10, 28, 35) were delivered by research assistants, and two (32, 38) employed a mixed approach.

Remote delivery methods were also frequent: 14 studies (4, 6, 10, 23–25, 27, 30, 31, 34, 35, 37, 41, 44) used telephone calls, text-message reminders, or smartphone apps. Most interventions began during pregnancy and continued postpartum, with a duration most frequent of 6 months (ranging from hospital-based single-session interventions to programs lasting 12 months) or 1 month. Counseling content routinely included hands-on guidance (latch and positioning), dietary advice for mothers, and motivational techniques. Twenty studies (69%) explicitly reported active learning strategies (open-ended questions, problem-solving exercises) rather than didactic lectures. Control groups were usually routine care, defined as standard health education or usual hospital discharge instructions, without structured breastfeeding counseling.

Overall, the interventions yielded positive breastfeeding outcomes. 16 out of 29 studies (55.2%) (3, 4, 6, 9, 10, 25–29, 32, 37, 40, 41, 43, 44) reported higher EBF rates or longer breastfeeding duration in the counseling groups versus controls. For example, Bueno-Gutiérrez et al. (9) found that formative, socio-ecological counseling increased 2-month EBF by 30% compared to usual care. Karaahmet and Bilgiç (37) reported that an online breastfeeding counseling program after cesarean delivery significantly improved EBF rates at 1 and 6 months, as well as maternal self-efficacy. In Bangladesh, a cluster trial by Ara et al. (3) showed peer counseling quintupled 5-month EBF (27 to 73%). Jerin et al. (10) combined hospital support with biweekly community phone counseling and observed EBF rise from 42% pre-intervention to 71% at 5 months (*p* < 0.001). Most studies that measured breastfeeding initiation also reported higher initiation rates in the counseling arms (data not shown). Secondary outcomes also improved. Many interventions raised maternal confidence and self-efficacy. For example, Fahim et al. (28) demonstrated a telephone-based educational intervention increased mothers’ scores on the Breastfeeding Self-Efficacy Scale (*p* < 0.001). Çetindemir and Cangöl (6) found a teach-back education program significantly improved mothers’ self-efficacy and latch (audible swallowing, nipple type, comfort, hold) scores at hospital discharge and 1-month follow-up. Several studies noted reductions in breastfeeding problems: for instance, mothers in counseling groups reported less nipple pain and better latch mechanics, and some studies saw lower maternal anxiety. A few trials even noted infant health benefits: one reported slightly higher infant growth (weight and length) in the counseling group (42). Table 1 summarizes each study’s setting, design, and key findings.

**Table 1:** Summary of included studies and main results.

Study (year, country)	Design/Participants	Intervention	Key findings
Bueno-Gutiérrez et al. (2021, Mexico) (9)	RCT; 80 mothers	Interpersonal counseling (socio- ecological model), 2- month follow-up	30% increase in EBF at 2 mo (*p* < 0.001); improved BF attitudes and self-efficacy in the intervention group.
Souza et al. (2020, Brazil) (40)	RCT; 104 postpartum women	BF education session vs. control	Higher EBF rates in intervention at 10, 30, 60 days postpartum (e.g., 86.5% vs. 44.2% at 60d) vs. control.
Karaahmet & Bilgiç (2022, Turkey) (37)	RCT; 151 primiparous women with cesarean section	Online BF counseling vs. routine care	BF rates at 1 and 6 mo were significantly higher in the counseling group; maternal self-efficacy scores were significantly higher in intervention.
Çetindemir & Cangöl (2024, Turkey) (6)	RCT; 100 mothers postpartum	Teach-back education vs. standard education	Teach-back group had significantly higher EBF success and self-efficacy at 1 mo (p < 0.001).
Rhodes et al. (2021, USA) (4)	Qualitative case study with 28 women (BHP program)	Peer counseling model for low-income minorities	BHP peer counselor program (home & hospital visits + phone) improved breastfeeding initiation and duration.
Rosen-Carole et al. (2022, USA) (22)	Pre-post intervention study with 70 1-month postpartum women	Prenatal BF toolkit vs. usual care	No major differences in intentions, but higher referral to support resources in the intervention.
Fahim et al. (2023, Iran) (28)	RCT; 64 pregnant teenagers	Midwife-led BF counseling vs. control	Significant increases in BF self-efficacy and improved BF behaviors in counseling group.
Shamsdanesh et al. (2023, Iran) (29)	RCT; 64 breastfeeding women	BF counseling + stress management vs. control	Counseling group showed reduced anxiety and depression (p < 0.001) and increased EBF rates.
Jerin et al. (2020, Bangladesh) (10)	Quasi-experimental study; 164 pregnant women	Hospital counseling + biweekly mobile support	EBF at 5 mo rose from 42 to 71% post-intervention (p < 0.001); overall 0–5mo EBF rose from 58 to 78%.
Karimi et al. (2022, Iran) (30)	Quasi-experimental study; 74 mothers with COVID-19	Continuous vs. intermittent counseling (video/ phone)	No difference in EBF continuation, but self-efficacy improved in continuous group.
Ara et al. (2018, Bangladesh) (3)	RCT; 350 mother-infant pairs	Peer counseling from the 3rd trimester to 6 months postpartum	Early initiation of BF (EIBF) was higher in the counseling group (89% vs. 77%, *p* = 0.005); EBF at 5mo 73% vs. 27% (p < 0.001).
Wable Grandner et al. (2022, Bangladesh) (33)	Qualitative study; mothers in rural communities, supported by CHWs	Storytelling by CHWs vs. standard messaging	Intervention increased male partner support.
Martinez-Brockman et al. (2020, USA) (23)	Qualitative study; 54 two-way text messages present in the LATCH study	Text message support group	Mothers reported greater motivation/knowledge, reduced anxiety, and a desire for family support.
Mohammadian et al. (2021, Iran) (31)	RCT; 65 mothers with late preterm infants	Continuous telephone counseling support for 14 days, conducted by BF specialists	BF self-efficacy in the intervention group increased from 33.18 to 53.48 (*p* = 0.001).
Rozga et al. (USA, 2015) (24)	Secondary analysis of programme data; 5,886 low-income women enrolled prenatally	Peer counseling with home visits, phone calls, and SMS support; provided by trained peer counselors	Longer duration of EBF; each additional contact with the peer counselor reduced the risk of breastfeeding discontinuation
Dalal et al. (2023, India) (42)	Quasi-experimental; 576 EBF infants from 0 to 14 weeks	Counseling on cross-cradle hold technique, proper latching, and effective milk transfer; prenatal and postnatal sessions, weekly home visits up to 14 weeks	67.3% infants in group gained ≥30 g/day vs. 39.9% in control at 2wks (*p* < 0.01); underweight prevalence lower in intervention group (5.3% vs. 16.7%)
Frith et al. (2017, Bangladesh) (34)	RCT; 3,186 pregnant and postpartum women who have experienced domestic violence and their infants, rural population	Personalized counseling with 8 sessions (2 during pregnancy, 6 postnatal); conducted by trained peer counselors	Intervention had EBF for an average of 135 days, vs. 60 days in the control; counseling neutralized the negative effect of domestic violence on breastfeeding duration
Flax et al. (2022, Nigeria) (44)	Interventional study; 1,200 pregnant women	BF counseling during their third trimester and at 6 and 24 weeks postpartum	Increase in EBF from 76 to 83% at 6 weeks and from 52 to 66% at 24 weeks; mothers who received messages were 1.7 times more likely to EBF.
Zunza et al. (2025 South Africa) (35)	RCT; 276 mothers living with HIV	MI with meetings at 2, 6, and 10 weeks postpartum; personalized weekly text messages; telephone follow-up; informational support on HIV and breastfeeding	NS effect on EBF at 24 weeks; lower incidence of infant hospitalization in the intervention group (NS).
Copeland et al. (2019, UK) (38)	Feasibility study, qualitative analysis; 70 mothers in three National Health Service maternity services	Peer counseling based on MI; prenatal meetings, contact within 48 h after birth, follow-ups every 2 days for 2 weeks, and continued follow-up until 6 weeks; supervised by MI experts and midwives	Intervention was considered acceptable, and health professionals felt it could be integrated with existing services.
Burns et al. (2020, Australia) (45)	Quali-quantitative descriptive survey study; 53 women in drop-in service with infants ranging in age from less than 1 week through to 12 months of age	Face-to-face peer counseling at a breastfeeding drop-in center; individualized breastfeeding support provided by trained peer counselors	95% of mothers rated the service as ‘very helpful’ or ‘extremely helpful’; 90% reported that helped them achieve their BF goals; lack of professional medical support identified as a barrier to BF success
Harari et al. (2018, USA) (25)	Feasibility study; 58 women at 18–30 weeks of gestation	WIC breastfeeding peer counseling; newborns followed up to 2 weeks post-partum	EBF rates at 2 weeks in intervention was 50% vs. 31.8% in the control arm (*p* = 0.197). Intervention group more likely to meet their BF goals (*p* = 0.06).
Addicks et al. (2019, USA) (26)	RCT; 81 Appalachian pregnant women	45-min single session of MI during pregnancy; techniques included change talk, open-ended questions, active listening, positive reinforcement	1 mo postpartum: 92.7% in the MI group were still BF vs. 79.5% in the control group (*p* = 0.040); MI significantly improved BF attitudes in primiparous women; NS on EBF or duration intentions
Franco-Antonio et al. (2022, Spain) (41)	RCT; 88 postpartum women	Brief MI intervention; single session (20–30 min) + booster call at 1 month; techniques included open-ended questions, active listening, change talk, problem solving, reinforcement of self-efficacy	Intervention: longer BF duration (11.06 weeks vs. 9.02 weeks, *p* = 0.013); increased BF self-efficacy (6.24-point increase); lower EPDS scores at 3 months (5.5 vs. 8, *p* < 0.05); lower PPD risk (11.9% vs. 30% in control).
Ahmadi et al. (2016, Iran) (32)	RCT; 124 mothers with premature infants	5 BF counseling sessions based on BASNEF model and GATHER approach.	Significant increase in EBF rates (72.6% vs. 16.1%); Improved lactation performance and neonatal weight gain; Enhanced maternal self-efficacy and confidence in BF
Scott et al., (2016, UK) (39)	Time series analysis; 5,790 mothers	Peer support intervention; one-to-one support; home visits and phone consultations; peer supporters trained in BF support	Significant increase in BF initiation (+0.55% per month) and at 2 weeks (+0.50% per month); no significant change at 6 weeks; higher maternal confidence and reduced anxiety
Nilsson et al. (2017, Denmark) (43)	RCT; 3,541 mothers	Structured BF counseling program in hospital (skin-to-skin, frequent breastfeeding, optimal positioning, increased father involvement, self-efficacy-based counseling)	Increased exclusive BF rates at 6 mo (6.6% vs. 5.1%, p = 0.04); reduced neonatal hospital readmissions (2.2% vs. 3.6%, p < 0.01); higher paternal involvement; no significant difference in maternal self-efficacy scores
Rotheram-Fuller et al. (2017, USA) (27)	RCT; 203 mothers	Home visiting program (postnatal contacts face to face or telephone 30 min) focus on BFsupport, maternal health, and social well-being; sessions structured around problem-solving, practical support, and goal setting	Longer breastfeeding duration at 3 months postpartum (60% vs. 50%); no significant difference in maternal weight loss or postpartum depression; improved infant growth (*p* < 0.07)
Tuthill et al. (2017, South Africa) (36)	RCT; 68 HIV-positive mothers	45-min individual counseling session based on the Information-Motivation-Behavioral Skills Model; Motivational Interviewing techniques focus on BF knowledge, motivation, and skills	No significant difference in EBF rates at 6 weeks (81.5% in both groups, *p* = 1.00); self-efficacy predicted BF duration; no significant impact on knowledge, motivation, or behavioral skills

RCT, randomized controlled trial; mo, months; BF, breastfeeding; EBF, exclusive breastfeeding; WIC, women, infants, and children program. WICPC, Women, Infants and Children Peer Counselors; d, days; BHP, Breastfeeding Heritage and Pride program; NS, non significative; CHW, Community Health Workers; BASNEF, Beliefs, Attitudes, Subjective Norms and Enabling Factors; GATHER, Greet clients, Ask clients about themselves, Tell clients about their choices, Help clients choose, Explain what to do, and Return for follow-up-on. LATCH, Lactation Advice Through Texting Can Help; MI, motivational interviewing; PPD, postpartum depression; EPDS, Edinburgh Postnatal Depression Scale.

## 4 Discussion

Our scoping review corroborates the hypothesis that nutritional counseling interventions can substantially improve breastfeeding outcomes across diverse settings. Consistent with WHO recommendations, studies employing structured counseling, whether via trained health workers or peer supporters, generally reported higher EBF rates and longer breastfeeding duration than standard care. For example, interpersonal counseling in Mexico yielded a 30% net gain in EBF at 2 months (9). Similarly, in Bangladesh, peer counseling interventions enhanced mothers’ ability to manage challenges and sustain breastfeeding at 5 months (10). These results underscore that it is the counseling process itself (knowledge transfer, skill-building, motivational support) that drives outcomes, not breastfeeding promotion alone. Moreover, counseling interventions often enhanced maternal confidence: targeted education (phone-based or teach-back methods) significantly raised self-efficacy scores (6, 9, 28, 30–32, 37, 41), which is known to be linked to sustained breastfeeding. In line with this, Ariani et al. highlighted that maternal breastfeeding self-efficacy is a principal determinant of successful exclusive breastfeeding, emphasizing the importance of strategies that empower mothers and strengthen their confidence (46). Recent evidence also underscores the importance of structured counseling in early postpartum care: a 2025 scoping review by Cavalcanti and colleagues found that interventions delivered during hospitalization in rooming-in care improve breastfeeding initiation and maintenance, with positive impacts on newborn health (8).

Several underlying factors influence mothers’ infant feeding decisions, which helps explain these findings. Mothers’ knowledge and beliefs are critical. A cross-sectional survey including 103 women (47) found that a better understanding of breastfeeding benefits and strong prenatal motivation were linked to choosing EBF. Higher maternal education and socioeconomic status were also associated with higher EBF rates (47, 48). For example, mothers with higher education levels are more likely to be exposed to accurate information on breastfeeding and to access professional support, which facilitates initiation and continuation of EBF. Similarly, socioeconomic resources can reduce stress and financial barriers, allowing mothers to dedicate more time and energy to exclusive breastfeeding. Our results are consistent with broader literature showing that demographic and socio-economic factors – such as education level, income, age and marital status – affect feeding choices (48). Social and cultural norms matter too. Common barriers include stigma around public breastfeeding, lack of community role models, and entrenched formula-feeding norms (49). For instance, the US Breastfeeding Heritage and Pride program (a peer counseling model) was designed after qualitative work in the Latino community identified obstacles like embarrassment feeding in public and limited social support (4). In our review, culturally tailored counseling (e.g., in the Mexico study) helped mothers overcome contextual barriers and stigma. Family and community support strongly influences decisions: several studies noted that involving husbands (43), partners or mothers-in-law improved outcomes, echoing findings that family support is key to breastfeeding success (4). In summary, a mother’s decision is shaped by the perceived health benefits for the baby and herself, her knowledge and confidence, and the support (or lack thereof) from her social environment (48). Effective counseling addresses many of these factors – it fills knowledge gaps, corrects misconceptions, and builds confidence – but broader socio-economic and cultural influences also play a role and may limit how much counseling alone can achieve.

Remote and low-cost strategies also proved to be effective. Jerin et al. showed that simple biweekly phone follow-ups could sustain EBF in settings lacking postpartum support (10). This corroborates Cochrane evidence that telephone support increases six-month EBF (7). Culturally tailored counseling (as in the Mexico study based on formative research) helped mothers overcome contextual barriers (pain, public feeding stigma). The family or community (e.g., husband, in-laws) inclusion in counseling, though not detailed in all studies, was noted as important in some contexts, echoing the literature on social support.

WHO’s 2018 guideline on breastfeeding counseling emphasizes evidence-based recommendations and trained providers (1). Several included studies noted that providers (nurses, midwives, peers) required specialized training in counseling techniques. Although most of the included studies describe counseling interventions delivered by midwives, general healthcare workers, or peer counselors. Interprofessional collaboration, such as through engagement with state-based perinatal quality collaboratives or other interprofessional perinatal partnerships, is essential to ensuring that families receive cohesive, comprehensive breastfeeding and lactation support (50). We echo calls in the literature to invest in standardized training and supportive supervision for breastfeeding counselors (1). Finally, ongoing monitoring and context-specific formative research (as done in Mexico) can help tailor counseling content to mothers’ needs, increasing effectiveness.

Oggero and colleagues’ systematic review on the effects of prenatal breastfeeding education found that such interventions can influence breastfeeding duration, particularly when psychological elements are included. It also noted that these interventions are more effective when accompanied by in-person postnatal support (51). Coimbra et al. highlighted the lack of research on counseling protocols in breastfeeding (BF). While existing studies have mentioned protocols, they predominantly rely on structured scripts for sessions. The review emphasized the necessity for an individualized approach, setting it apart from health education, guidance, or clinical management of breastfeeding (52).

Future research should aim to standardize definitions of counseling interventions, compare different counseling models head-to-head, and assess long-term and cost-effectiveness outcomes. Randomized trials with longer follow-up and in underrepresented settings would help refine best practices. Exploring digital platforms (apps, text messages, phone calls) and engaging family members in counseling are promising avenues.

The techniques employed, such as active listening, problem solving, open-ended questioning, and practical support, are fully consistent with the relational approach typical of NC, especially when applied to the breastfeeding context.

According to recent definitions, NC is an interactive and collaborative process, person-centered, and aimed at improving behaviors and habits related to nutrition, regardless of the specific professional role involved (14, 53).

However, a critical issue emerges: the dietitian is rarely included in the teams supporting breastfeeding, despite their specific expertise in the nutritional field. It would therefore be desirable to promote a multidisciplinary approach that includes, alongside midwives, physicians, and peer counselors, the figure of the dietitian as a nutrition professional capable of providing specialized guidance on maternal and infant nutrition during breastfeeding.

In this perspective, NC should be promoted as an integral part of the overall care of the mother, going beyond the mere promotion of breastfeeding and encompassing nutritional education, motivational support, and the personalized adaptation of dietary recommendations.

Nevertheless, nutritional support during pregnancy and breastfeeding is crucial to ensuring optimal health outcomes for both mother and child. In particular, adherence to the Mediterranean Diet (MD) during this period has been associated with improved maternal psychological well-being, healthier postpartum weight management, better glucose metabolism, and a more favorable fatty acid profile in human milk (HM) (54). In this context, nutrition education based on the principles of the MD can accompany NC during lactation, offering mothers a nutrient-rich and sustainable dietary model to support both their own well-being and that of their infants.

The evidence gathered in this review shows that many general counseling techniques are already effectively used in breastfeeding support pathways. However, the systematic inclusion and recognition of the dietitian would ensure a truly nutritional and personalized approach, addressing a gap that is currently evident in the international literature.

Certainly, some challenges and limitations emerge. There is no standardized definition of “nutritional counseling” in the breastfeeding context, leading to heterogeneity in intervention content. Some studies highlighted that if counseling is reduced to didactic education only, its relational value could be lost (4, 55). We observed that multifaceted approaches (combining technical advice with motivational interviewing and emotional support) were more successful. The studies also varied in quality and design; only a few were large-scale trials. Moreover, follow-up durations differed, making comparison difficult. Not all interventions recorded statistically significant results (e.g., some small pilots showed trends but no significance), possibly due to limited power. Cultural and health-system factors also influenced outcomes: an intervention effective in one region may need adaptation elsewhere.

## 5 Conclusion

This scoping review underscores that targeted counseling strategies are generally effective in promoting EBF and improving maternal confidence. Personalized, empathetic counseling, whether delivered face-to-face or remotely, can significantly raise EBF rates and help mothers overcome feeding challenges. For practice, our findings suggest integrating structured counseling into routine maternal care (prenatal visits, immunization clinics, community programs), utilizing both professional and peer supporters. Policymakers should ensure that such interventions are culturally adapted and that providers receive proper counseling skills training. Ultimately, investing in high-quality counseling as part of breastfeeding support programs may help reach global breastfeeding targets and improve child and maternal health. Future research should clarify the definition of counseling in this field and explicitly evaluate nutritional counseling approaches, including the role of dietitians within multidisciplinary breastfeeding support teams.

## Data Availability

The original contributions presented in the study are included in the article/supplementary material, further inquiries can be directed to the corresponding author.
